# The brain subcortical white matter and aging: A quantitative fractional anisotropy analysis

**DOI:** 10.1590/S1980-57642009DN30300009

**Published:** 2009

**Authors:** Eliasz Engelhardt, Denise Madeira Moreira, Jerson Laks

**Affiliations:** 1 Coordinator, Cognitive and Behavioral Neurology Unit - INDC/UFRJ, Rio de Janeiro RJ, Brazil.; 2 Coordinator, Neuroimaging Unit - INDC/UFRJ, Rio de Janeiro RJ, Brazil.; 3 Radiologist, Pró-Cardíaco Hospital/RJ.; 4 Coordinator, Center for Alzheimer’s Disease - CDA-IPUB/UFRJ.; 5 Medical Sciences Faculty, UERJ, Rio de Janeiro RJ, Brazil.

**Keywords:** White matter, corpus callosum, fractional anisotropy, aging

## Abstract

To study the integrity of hemispheric subcortical white matter by comparing normal young and elderly subjects using quantitative fractional anisotropy (DTI-FA).

**Methods:**

Subjects of two different age groups (young=12, elderly=12) were included. MR - GE Signa Horizon - 1.5T scans were performed. Cases with Fazekas scores =3 were assessed on FLAIR sequence. Standard parameters for DTI-FA were used. ROIs were placed at various sites of the subcortical white matter, and the genu and splenium of the midline corpus callosum. Analysis was performed using Functool. Statistics for anterior and posterior white matter, as well as the genu and splenium were compared between the groups. The study was approved by the Ethics Committee of IPUB-UFRJ and informed consent obtained.

**Results:**

DTI-FA showed lower anisotropy values in the anterior region (subcortical white matter and genu), but not in the posterior region (subcortical white matter and splenium), in elderly normal subjects compared to young subjects.

**Conclusion:**

The results may represent loss of integrity of anterior (frontal) white matter fibers in the elderly subjects. These fibers constitute important intra- and inter-hemispheric tracts, components of neural networks that provide cognitive, behavioral, motor and sensory integration. The loss of integrity of the anterior segments of the studied fiber systems with ageing, represents a disconnection process that may underlie clinical manifestations found in elderly subjects such as executive dysfunction.

## Abstract (pt)

Estudar a integridade da substância branca hemisférica subcortical, comparando sujeitos normais jovens e idosos, com anisotropia fracionada quantitativa (DTI-FA).

**Métodos:**

Foram incluídos sujeitos de dois grupos etários (jovem=12, idoso=12). Obtidas imagens de MR - GE Signa Horizon - 1.5T. Escore de Fazekas =3 avaliado na sequência em FLAIR. Utilizados parâmetros padrão para DTI-FA. ROIs colocados em locais variados da substância branca subcortical, e no joelho e esplênio do corpo caloso na linha média. Análise com Functool. Estatística para comparar a substância branca anterior e posterior entre os grupos. Aprovado pela Comissão de Ética do IPUB-UFRJ, consentimento informado obtido.

**Resultados:**

DTI-FA mostrou redução dos valores de anisotropia na região anterior (substância branca subcortical e joelho), porém não na região posterior (substância branca subcortical e esplênio), nos sujeitos normais idosos em comparação aos jovens.

**Conclusão:**

Os resultados podem ser considerados como representando perda da integridade das fibras da substância branca anterior (frontal) no sujeitos do grupo idoso. Tais fibras constituem os importantes feixes intra- e inter-hemisféricos, componentes de redes neurais relacionadas com integração cognitiva, comportamental, motora e sensorial. A perda da integridade com o envelhecimento dos segmentos anteriores dos sistemas de fibras estudados representa um processo de desconexão que pode estar subjacente a manifestações clínicas, como a disfunção executiva, eventualmente encontradas em sujeitos idosos.

The subcortical white matter makes up around half of the human brain volume. It is responsible for the interconnection of cortical and subcortical areas, participating in the constitution of the wide neural networks related to a host of motor, sensory, cognitive, and behavioral functions. This white matter is composed of fiber bundles which are classified as projection, associative and commissural tracts. The main projection systems are constituted by the cortico-bulbar, cortico-spinal, and cortico-pontine fibers. The associative bundles include the superior and inferior longitudinal tracts, the superior and inferior occipito-frontal tracts, and the limbic uncinate fascicle, cingulum, and fornix while commissural fibers constitute the large corpus callosum and the anterior comissure. These tracts of fibers spread out anteriorly, posteriorly, and laterally, where they intermingle in the centrum semiovale and with the fibers of the corona radiata.^1-3^

Structural imaging techniques (computer tomography and magnetic resonance) reveal the subcortical white matter in a clear yet homogeneous way.^4-7^ The recently developed diffusion tensor imaging (DTI) technique offers a new opportunity to evaluate the brain white matter architecture in a qualitative and quantitative manner, both in normal and pathological states. A detailed analysis of the white matter with DTI is possible given two of its features – mean diffusivity and the fractional anisotropy (FA). Currently, the most widely used measure of anisotropy is DTI-FA that allows quantification, where the values obtained represent an average of the sampled fibers in a given region of interest (ROI). It is a highly sensitive but fairly nonspecific biomarker of neuropathology and microstructural architecture of white matter and is generally considered a marker of its integrity.^8-9^ Several studies demonstrated that the organization of white matter fiber bundles is the basis for DTI-FA. The myelin appears to influence its measures, as well as axonal damage and loss. The parallel organization of white matter fiber bundles is the basis for anisotropic diffusion, whereas myelin appears to modulate the amount of anisotropy.^8^ DTI-FA appears to be the most sensitive imaging parameter to determine age-related white matter damage, and the strong relationship of such damage with this parameter suggests that axonal damage may be important in age-related cognitive decline.^10^

Analysis of lesions identified by neuroimaging and verified neuropathologically has shown that low DTI-FA values are indicative of axonal damage and demyelination.^8-9^ However, analysis of regions visually identified as unaffected, may also show similar derangement of the microarchitecture of the white matter.^11-12^ Changes may also be seen upon comparing brains of younger with older subjects, showing the effect of age on white matter.^5-6^

The objective of this study was to describe the subcortical white matter in normal subjects by comparing a young group with an elderly group of subjects using quantitative fractional anisotropy (DTI-FA). The relevance of such a study lies in the evaluation of structural age-related changes, which may provide a better understanding of pathophysiological aspects of possible age-related cognitive changes. In addition, such quantitative structural age-related change analysis in normal subjects may serve as a reference standard against which individuals with neuropathological disorders may be compared.

## Methods

### Subjects

The present study included two samples of normal subjects, one young group and one elderly group. The rationale for the choice of the groups was based on structural and cognitive data, as well as on clinical aspects. The longitudinal volumetric assessments showed a declining curve beginning around the age of 30 progressing clearly after the age of 60 years.^13^ Longitudinal cognitive evaluation demonstrated that age-related cognitive decline begins in healthy young adults at around the age of 30 years, and proceeds in several aspects thereafter.^14^ In the clinical setting, the 30-year-old age bracket is considered paradigmatic for comparisons in studies on cognitive decline.^15^ The subjects included in this investigation had no cognitive or behavioral complaints at the time of the evaluation. The characteristics of the samples are shown in Table 1.

**Table 1:** Characteristics of the samples.

	Young	Elderly
N	12	12
Sex (m/f)	4/8	5/7
Age (years)	30.6±5.7	74.8±5.1
(range)	(24-40)	(66-82)
Education (years: Mean ±sd)	12.5±3.71	12.4±2.43
MMSE^a^ (score: Mean ±sd)	28.8±1.11	27.4±2.70
CDR^b^ (score)	0	0
Hachinski^c^ (score)	0.17±0.58	0.92±0.79
Fazekas^d^ (score)	0.17±0.39	2.0±0.85

a MMSE, Mini-Mental State Examination (short cognitive screening tool)^16^;

b CDR, Clinical Dementia Rating scale (global severity stages from 0 to 3)^17^;

c Hachinski, ischemic score (clinical assessment of vascular risk)^18^;

d Fazekas, white matter lesion scale (severity from 0 to 6)^19^.

### Techniques

A standard series of MR scans of the brain, complemented with DTI acquisitions, was obtained for the two samples on a 1.5T GE Signa Horizon device. Axial plane fluid-attenuated inversion recovery (FLAIR) sequence scans were examined to evaluate the presence of white matter lesions, which were classified according to Fazekas’s scoring system.^19^ Only cases with score ≤3 (visual assessment) were included. The scoring was performed by two of the authors (DMM and EE) in consensus.

The parameters for the DTI-FA acquisition employed in the present study were as follows: TR/TE=10000/89.1 msec, matrix=128×128, FOV=30×24 mm, NEX=1, b=1000 sec/mm^2^, slice thickness=5 mm, number of slices=30 without gap, being in-line with values found in international studies on the theme.

Circular ROIs of 60 mm^2^ were placed at 14 symmetrical regions of both hemispheres on two axial planes (basal ganglia and supracallosal levels, parallel to the AC-PC line) of the DTI-FA maps (total number of ROIs=168 for each group). For statistical analysis, the ROIs were divided into anterior (frontal) and posterior (temporo-parieto-occipital) regions. It should be noted that the anterior and posterior groups of ROIs were positioned to measure the intermingled fibers of the anterior segments of the inferior and superior occipito-frontal tracts and uncinate fascicle, the superior longitudinal tract, besides other fibers of the anterior corona radiata, and also the lateral spread of the anterior segment of the corpus callosum, and to measure posterior segments of the inferior occipito-frontal and inferior and superior longitudinal tracts, besides other fibers of the superior and posterior corona radiata, as well as the lateral spread of the posterior segment of the corpus callosum. Additionally, ROIs were placed on the genu and the splenium of the midline corpus callosum on one axial plane (basal ganglia level, parallel to the AC-PC line) of the DTI-FA maps (total number of ROIs=24 for each group), where callosal fibers alone could be evaluated^1,20^ (Figure).

**Figure:**
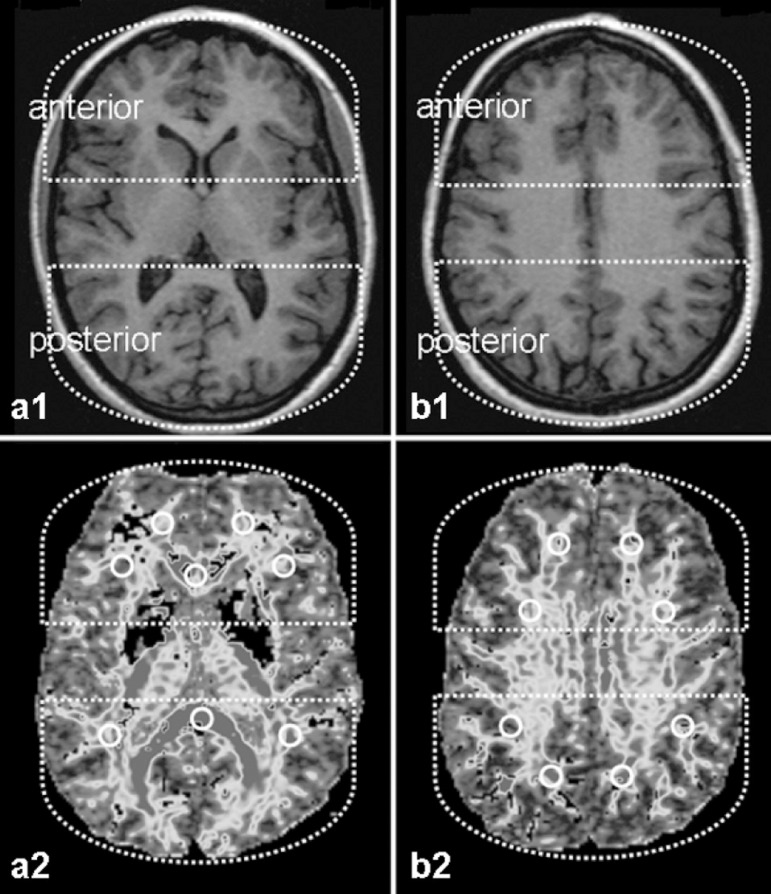
MR scans (axial sections at basal ganglia and supracallosal levels) in 3DT1 sequence (a1 and b1) for topographical reference, and DTI-FA maps at the same levels (a2 and b2) (in black and white). The ROIs are shown in the DTI-FA maps, localized in the subcortical white matter (anterior and posterior regions circumscribed by broken lines), and corpus callosum (genu and splenium).

The DTI-FA maps were analyzed on an ADW 4.3 Work Station using the Functool 4.5.3(GE Medical Systems). The averaged values of the subcortical white matter ROIs were pooled for anterior (frontal) and posterior (temporo-parieto-occipital) regions, and the corpus callosum ROIs into genu and splenium sites. Statistical analysis^21^ was performed to compare intra-sample and inter-sample values of anterior and posterior regions of the subcortical white matter, and of genu and splenium of the corpus callosum. Basic statistics were applied, and mean±sd calculated for the anterior and posterior groups of values of the subcortical white matter, and the same for the genu and splenium of the corpus callosum of each sample. Student’s t test was used to assess statistical differences between the studied regions.

### Ethics

The present study is part of a larger project on Vascular Cognitive Disorder, approved by the Ethics Committee of IPUB-UFRJ. Informed consent was obtained from the participants before they embarked on the study.

## Results

The DTI-FA data on subcortical white matter and the corpus callosum of the young and old groups are depicted in Table 2.

**Table 2:** Subcortical white matter and corpus callosum. Results of quantitative FA in young vs. elderly.

Regions	ROIsn per group	FA units (mean±sd)		p-value^‡^
		Young group	Elderly group	
Subcortical white matter				
Anterior*	96	0.3629±0.09	0.3122±0.05	0.0001
Posterior**	72	0.3997±0.07	0.3937±0.09	0.6559
p-value^‡^	-	0.0045	0.0001	-
Corpus callosum				
Genu	12	0.6861±0.08	0.6041±0.05	0.0064
Splenium	12	0.7397±0.06	0.7230±0.04	0.4310
p-value^‡^	-	0.1150	0.0001	-

* frontal;

** temporo-parieto-occipital;

‡ Student p-value.

The anterior region of the subcortical white matter in the elderly showed significantly reduced DTI-FA values in comparison to the young group (inter-sample), but no difference between values of the posterior regions. Significantly lower values were observed in the anterior white matter compared to the posterior region in both groups, although this difference was more significant in the elderly (intra-sample). The DTI-FA values for the genu, but not the splenium, were significantly lower in the elderly in comparison to the young group (inter-sample). Values for the genu were found to be significantly lower than those for the splenium in the elderly group, but not the young group (intra-sample).

In sum, the inter-sample comparison of the anisotropy values showed significantly lower values in the elderly than the young group for anterior subcortical white matter and the genu, but not between the values of the posterior white matter and the splenium. The intra-sample comparison revealed significantly lower values for the anterior subcortical white matter in relation to the posterior in both groups, reaching greater significance in the elderly. For the corpus callosum, the anisotropy values of the genu in comparison to the splenium were significantly reduced in the elderly, but not in the young group.

## Discussion

The present data showed changes in DTI-FA values between young and elderly groups, both in subcortical white matter (anterior [frontal] and posterior [temporo-occipito-parietal] regions), and midline corpus callosum (anterior [genu] and posterior [splenium] segments).

The inter-sample measures of the anterior subcortical white matter (represented by anterior segments of several anteroposterior tracts, subcortico-cortical fibers, and the lateral spread of the corpus callosum, as well as of the genu), showed significantly lower anisotropy in the elderly compared to the young group. This reduction was not observed in relation to the posterior subcortical white matter (represented by posterior segments of several anteroposterior tracts, subcortico-cortical fibers, and the lateral spread of the corpus callosum, and the splenium). On intra-sample data comparisons, significantly lower values were seen for anterior white matter than for posterior regions in both groups, with more significant difference in the elderly. Thus, the present data demonstrate that, in terms of anisotropy values, the anterior white matter is more vulnerable to aging than posterior regions. This finding confirms the suggestion of an anterior-to-posterior gradient described previously.^22-23^

Studies on changes in subcortical white matter and corpus callosum with aging have been published by several international groups, although no reports were found in the national literature. These studies related to ageing, reported differential axonal loss and demyelination of the fibers that constitute the several tracts of subcortical white matter, including the corpus callosum, with changes affecting the anterior regions to a greater degree than the posterior region.^5,6,22,24-28^ These results are comparable to those of the present study.

The associative subcortical white matter tracts establish the long intra-hemispheric connections, with information traveling between anterior and posterior regions of the hemispheres, while the corpus callosum is the main neocortical commissure, and forms most inter-hemispheric connections.^1,26,29,30-32^ These fiber systems participate in the large neural networks that support (bi)-hemispheric activities, and underpin the complex cognitive, behavioral, motor and sensory functions. The disruption of these networks may be related to impairment of neural integration through disconnection mechanisms, one of the proposed causes of cognitive changes seen in pathological states and ageing.^10,12,29-30,33-34^

Thus, it is conceivable that the subcortical white matter tract and corpus callosum damage that occurs predominantly in anterior regions in elderly subjects, as observed in the present and other studies, may be related to anterior disconnection manifestations.^10,33-38^ Considering their relation to the anterior high-level integrative regions, such disconnections are of importance in providing a structural basis for the vulnerability and eventual impairment of the complex executive function cognitive domain.^24,37-45^

Quantitative DTI-FA studies that assess structural age-related changes may be help provide a better understanding of pathophysiological aspects of cognitive manifestations related to normal aging. Additionaly, such studies may serve as a standard for comparison with various structural-related brain disorders.^7,21^

## Conclusion

The ageing process of the brain subcortical white matter and corpus callosum correlates with changes in anisotropy values. These changes may presently be revealed by quantitative DTI-FA, an in vivo marker of fiber integrity. DTI-FA appears to be the most sensitive imaging parameter for determining age-related white matter damage. Furthermore, the frontal regions seem to be more vulnerable to aging in comparison to posterior regions.

The brain regions of normal subjects studied (anterior and posterior subcortical white matter, associative and commissural) showed lower DTI-FA values in the anterior region (subcortical white matter and genu), but not in the posterior region (subcortical white matter and splenium) in the elderly compared to the young group. These findings highlight the vulnerability of the anterior region and reinforce the notion of an anterior-posterior gradient of fiber loss.

The subcortical white matter tracts participate in the constitution of the wide neural networks which form the basis of cognitive, behavioral, motor and sensory integration. Loss of integrity in the anterior segments of the studied fiber systems with ageing, represents a disconnection process that may underlie clinical manifestations found in the elderly subjects such as executive dysfunction.
